# Neuritis ossificans: rare cause of sciatica

**DOI:** 10.11604/pamj.2016.25.170.9937

**Published:** 2016-11-16

**Authors:** Salah Bellasri, Cherif El Asri

**Affiliations:** 1 Service d’Imagerie Médicale, Hôpital Militaire, Faculté de Médecine et de Pharmacie, Université Mohammed V, Rabat, Morocco; 2 Service de Neurochirurgie, Hôpital Militaire, Faculté de Médecine et de Pharmacie, Université Mohammed V, Rabat, Morocco

**Keywords:** Neuritis ossificans, sciatica, lumbar spine

## Image in medicine

A 35-year old male, presented with 3 months history of intermittent sciatica involving the right lower extremity. There was no history of trauma or intra muscular injection. Results of neurological examination included a negative right sided Lasègue’s sign, an absent right Achilles tendon reflex, and decreased pinprick sense in the right S-1 distribution. Non-steroid anti-inflammatory drugs (NSAID) and myo-relaxant were prescript. Six weeks after his last visit to the neurosurgery outpatient clinic, the patient came back because during this period his condition did not improve. A computerized tomography (CT) scan demonstrated a localized calcification of the right S1 root.

**Figure 1:**
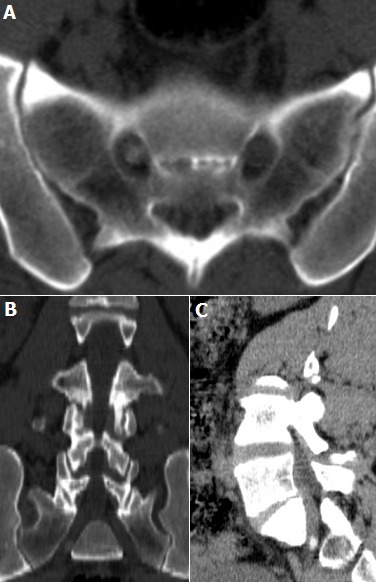
(A) axial CT slice presented in bone window: showed a round shaped calcification of the right S1 root; (B) coronal oblique reconstructions in bone window: showed a round shaped calcification of the right S1 root; (C) sagittal oblique reconstructions in soft tissue window: showed a round shaped calcification of the right S1 root

